# Effects of Climate Change and Fencing on Forage Nutrition Quality of Alpine Grasslands in the Northern Tibet

**DOI:** 10.3390/plants12183182

**Published:** 2023-09-06

**Authors:** Guangyu Zhang, Erfu Dai, Dawaqiongda, Luobu, Gang Fu

**Affiliations:** 1 Lhasa Plateau Ecosystem Research Station, Key Laboratory of Ecosystem Network Observation and Modeling, Institute of Geographic Sciences and Natural Resources Research, Chinese Academy of Sciences, Beijing 100101, China; zhanggy.19b@igsnrr.ac.cn (G.Z.); daief@igsnrr.ac.cn (E.D.); 2 Zhongba County Agriculture and Animal Husbandry Comprehensive Service Center, Zhongba County 858800, China; zbxnmj@126.com (D.); 13628922893@163.com (L.)

**Keywords:** biodiversity, acid detergent fiber, crude ash, crude protein, dissolvable total sugar, ether extract, neutral detergent fiber, alpine regions

## Abstract

How climate change and fencing will affect forage nutrition quality of alpine grasslands is still unknown in the Northern Tibet. Here, we reported the effects of climate change and fencing on forage nutrition quality (i.e., CP: crude protein, ADF: acid detergent fiber, NDF: neutral detergent fiber, Ash: crude ash, EE: ether extract and DTS: dissolvable total sugar) in alpine grasslands across the Northern Tibet based on a transect survey dataset from 2018. Over the whole survey transect, fencing reduced the NDF content by 5.15% and the EE content by 15.79%, but did not affect forage nutrition quality (*R*^2^ = 0.04, *p* = 0.389). Air temperature and precipitation explained 24% and 8% of variation in the CP content under the fencing conditions, respectively. Precipitation explained 22% of variation in the NDF content under the fencing conditions. The CP content decreased and increased exponentially with increasing air temperature under the fencing and grazing conditions, respectively. The NDF content showed logarithmic and negative relationships with precipitation under the fencing and grazing conditions (−8.45 vs. −6.68lnNDF). The response of the CP content to fencing showed negative relationships with temperature and the response of AGB to fencing, but showed a positive relationship with precipitation. The CP and DTS contents showed negative relationships with AGB under the fencing and grazing conditions. In contrast, the ADF content showed a positive relationship with AGB. The response of AGB, SR and community composition to fencing explained 11%, 56% and 35% of variation in the response of forage nutrition quality to fencing, respectively. Therefore, climate change may not always have adverse effects on forage nutrition quality, whereas fencing may not always have favorable effects on forage nutrition quality. Fencing and climate change can have an interactive effect on forage nutrition quality. Fencing can alter the temperature and precipitation sensitivities of forage nutrition quality. In colder and wetter regions, the forage nutrition quality may be more responsive to fencing. There may be a trade-off between forage nutrition quality and quantity. Compared to the change in AGB caused by fencing, the changes in species α-diversity and community composition caused by fencing can have greater effects on the response of forage nutrition quality to fencing. Local climate conditions and the trade-offs between forage nutrition quality and biomass should be considered when evaluating the effects of fencing on the restoration of degraded grassland plants.

## 1. Introduction

Forage nutrition quality of grasslands, as an important aspect of grassland evaluation, not only affects the growth and development of livestock, but also affects the yield and quality of livestock products [1,2]. Moreover, forage nutrition quality of grasslands can play a key role in the young recruitment and population dynamics of wild herbivores [3,4]. Generally, the most common forage nutrition quality variables include protein, fiber and others [5,6]. Crude protein (CP) is positively correlated with the nutrition quality of herbage [7,8]. Acid detergent fiber (ADF) is negatively correlated with herbage digestibility [8,9]. Neutral detergent fiber (NDF) is negatively correlated with herbage palatability [5,9]. Increasing protein content and decreasing cellulose content are important ways to improve forage nutrition quality [10,11,12]. Global warming is widely recognized as an indisputable fact, and meanwhile global precipitation has changed significantly [13,14,15]. Fencing, as one of the most common human constructions, is considered an important way of restoring degraded grasslands [16,17]. Therefore, understanding the responses of forage nutrition quality of grasslands to climate change and fencing is crucial for predicting future changes in the ecosystem services of grasslands and the protection of wild herbivores [2,5].

A growing number of studies have investigated the impacts of fencing and/or climate change (warming, precipitation change) on grasslands at various spatial and temporal scales [18,19,20,21,22]. However, several non-completely mutually nonexclusive debates remain. First, on one hand, previous studies generally have focused on the effects of climate change and/or fencing on the characteristics of grassland quantity (e.g., grassland productivity and species diversity) rather than the characteristics of grassland nutrition quality [23,24,25]. On the other hand, most studies related to forage nutrition quality in grasslands are conducted at the plant species or organ levels rather than the community/ecosystem/landscape levels [10]. Moreover, both climate change and fencing can have negligible, positive or negative [2,26] effects on the forage nutrition quality of grasslands. These diverse effects may be attributed to their different plant community compositions and climatic conditions [2,5]. These findings imply that the general tendency of the response of forage nutrition quality to climate change and fencing in native grasslands remains unclear. Second, many studies have investigated the relationships between forage nutrition quality and aboveground biomass (AGB) in grasslands [1,27]. They found that forage nutrition quality is negatively correlated with AGB to some extent in grasslands [1,27]. However, few studies have examined the relationships between forage nutrition quality and plant species α-diversity and community composition at the landscape level in natural grasslands. These findings imply that it remains unclear how plant species α-diversity and community composition affect forage nutrition quality in natural grasslands. Moreover, it is also still unclear which one of AGB, plant species α-diversity and community composition has the closest correlation with forage nutrition quality. Therefore, more studies are needed to better resolve the two questions mentioned above.

As an important region of the National Ecological Safety Construction, Northern Tibet is mainly occupied by various alpine grasslands (e.g., alpine meadows, alpine steppes, alpine desert steppes) [28,29]. These diverse alpine grassland ecosystems are important components of global alpine ecosystems [20,30]. Alpine grasslands in the Northern Tibet have suffered and will continue to suffer from both climate change and human activities [1,13]. On one hand, the Northern Tibet, as one sensitive region to climate change, has been becoming and will continue to become warmer and wetter [13]. On the other hand, the grasslands of the Northern Tibet have been locally degraded since 2000 [13,31,32]. Fencing and other ecological protection measures have been used to restore degraded grasslands under such a background. Only a few studies have tried to examine the effects of fencing and/or climate change on forage nutrition quality in the Northern Tibet grasslands [2,5]. However, these few studies have mainly discussed the relationships between forage nutrition quality and climate factors. Moreover, only one study has tried to investigate the relationships between forage nutrition quality and plant species α-diversity and community composition in three alpine meadow sites of the Northern Tibet along an elevation gradient from 4300 m to 4700 m [8]. This fact means that the spatial scale of the study mentioned above is relatively small [8]. There is also still an absence of studies on the relationships between forage nutrition quality and plant species α-diversity and community composition on a larger spatial scale. No studies have investigated the relationships between forage nutrition quality and species richness and plant community composition in alpine grasslands across the Northern Tibet. In addition, these studies do not investigate the relationship between forage nutrition quality and forage yield across the Northern Tibet. Therefore, more studies are needed to better examine whether there are trade-off relationships between forage nutrition quality and AGB across the Northern Tibet.

In this study, we reported the effects of fencing and climate change on forage nutrition quality across the 13 alpine grassland sites in the Northern Tibet. The main objectives of this study were to examine (1) whether there were trade-offs between forage nutrition quality and AGB and plant species richness, (2) whether climate change always decreased forage nutrition quality and fencing altered the sensitivity of forage nutrition quality to climate change, and (3) whether forage nutrition quality was more responsive to fencing in wetter and warmer regions in alpine grasslands across the Northern Tibet. Based on previous studies [1,27], we hypothesized that forage nutrition quality had trade-offs with AGB and plant species α-diversity. Based on other previous studies [2,8], we hypothesized that forage nutrition quality was more sensitive to fencing in wetter regions but not correlated with temperature. Based on some previous studies [2,5], we hypothesized that climate change did not always decrease forage nutrition quality, and fencing can alter the response of forage nutrition quality to climate change.

## 2. Results

### 2.1. Trade-Off between Forage Quantity and Nutrition Quality

**Figure 1:**
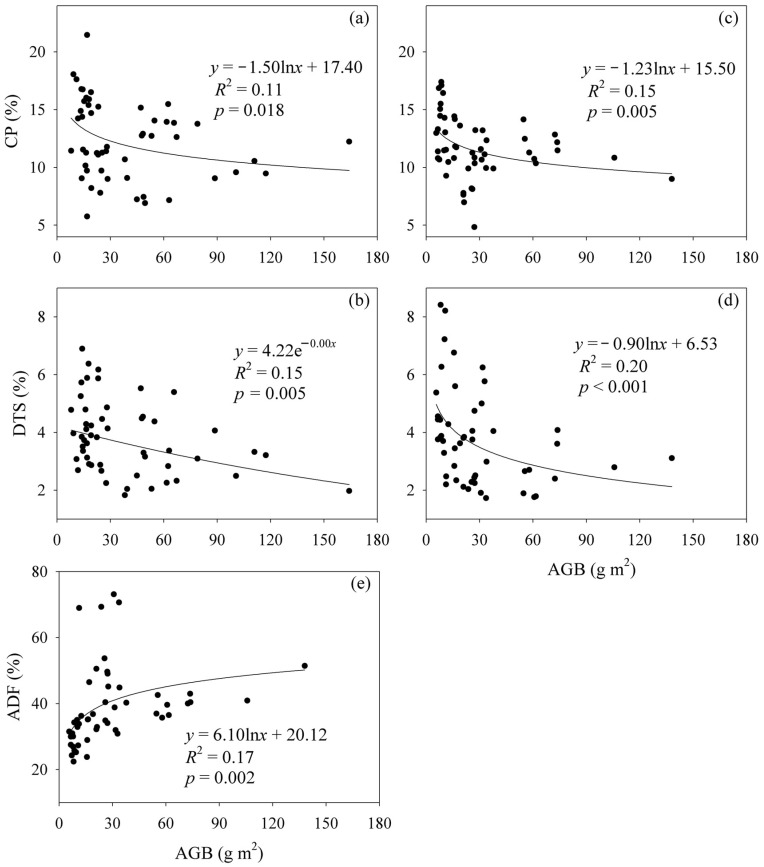
Relationship (**a**) between crude protein (CP) content and aboveground biomass (AGB) under the fencing conditions, (**b**) between dissolved total sugar (DTS) content and AGB under the fencing conditions, (**c**) between the CP content and AGB under the grazing conditions, (**d**) between DTS content and AGB under the grazing conditions and (**e**) between acid detergent fiber (ADF) content and AGB under the grazing conditions.

**Figure 2:**
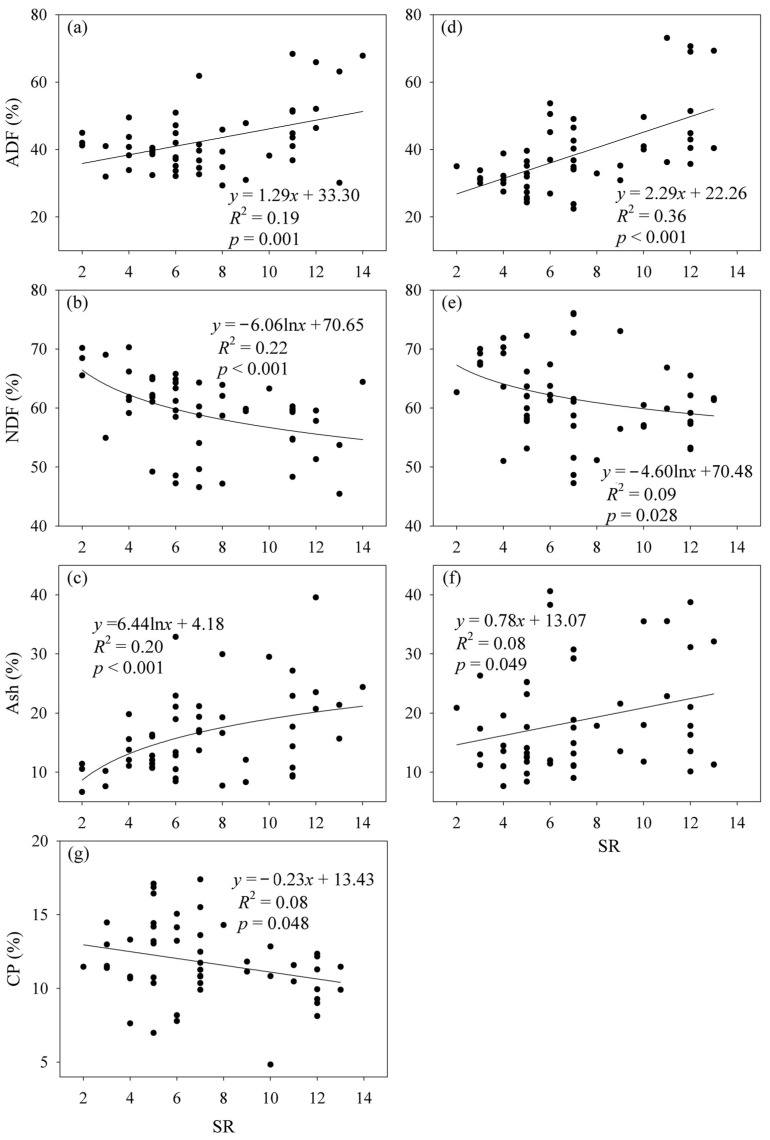
Relationship (**a**) between acid detergent fiber (ADF) content and species richness (SR) under the fencing conditions, (**b**) between neutral detergent fiber (NDF) content and SR under the fencing conditions, (**c**) between crude ash (Ash) content and SR under the fencing conditions, (**d**) between ADF content and SR under the grazing conditions, (**e**) between NDF content and SR under the grazing conditions, (**f**) between Ash content and SR under the grazing conditions and (**g**) between crude protein (CP) content and SR under the grazing conditions.

The CP and DTS contents showed negative relationships with AGB under the fencing and grazing conditions (Figure 1). In contrast, the ADF content showed a positive relationship with AGB under grazing conditions (Figure 1). The ADF and Ash contents showed positive relationships with SR, while the NDF content showed a negative relationship with SR under the fencing and grazing conditions (Figure 2). The CP content decreased with increasing SR under grazing conditions (Figure 2). Plant community composition showed correlations with the ADF, NDF and DTS contents under the fencing conditions, and with the CP, ADF, Ash and EE contents under grazing conditions (Table S1). The data matrix of CP, ADF, NDF, Ash, EE and DTS contents was closely correlated with SR and plant community composition under the fencing and grazing conditions (Table S2).

### 2.2. Climate Change Effects

**Figure 3:**
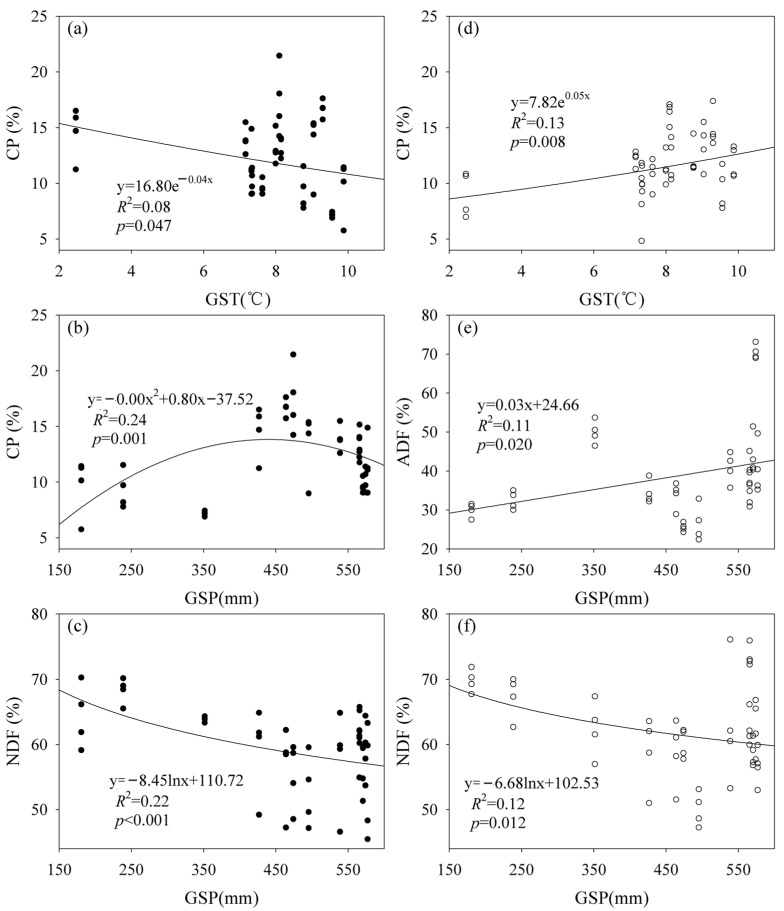
Relationship (**a**) between crude protein (CP) content and growing season temperature (GST) under the fencing conditions, (**b**) between CP content and growing season precipitation (GSP) under the fencing conditions, (**c**) between neutral detergent fiber (NDF) content and GSP under the fencing conditions, (**d**) between CP content and GST under the grazing conditions, (**e**) between acid detergent fiber (ADF) content and GSP under the grazing conditions, and (**f**) between NDF content and GSP under the grazing conditions.

Both SR and AGB increased with increasing GSP under the fencing and grazing conditions (Figure S1). Plant community composition showed correlations with GST (*R* = 0.27, *p* = 0.001) and GSP (*R* = 0.40, *p* = 0.001) under the fencing conditions, and GST (*R* = 0.37, *p* = 0.001) and GSP (*R* = 0.41, *p* = 0.001) under the grazing conditions. The CP content showed a negative relationship with GST, and a quadratic relationship with GSP under the fencing conditions (Figure 3). By contrast, the CP content showed a positive relationship with GST under the grazing conditions (Figure 3). The NDF content decreased with increasing GSP under the fencing and grazing conditions, while the ADF content increased with increasing GSP under the grazing conditions (Figure 3). The data matrix of GST and GSP showed correlations with plant community composition and the SR, CP and NDF contents under the fencing conditions, and with plant community composition and SR under the grazing conditions (Table S3).

**Figure 4:**
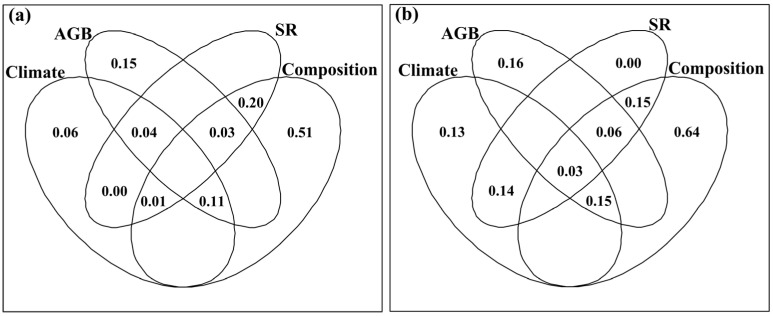
Venn plots of variation partitioning analysis, showing the shared and exclusive effects of climatic factors, aboveground biomass (AGB), species richness (SR) and community composition on the data matrix of crude protein, acid detergent fiber, neutral detergent fiber, crude ash, ether extract and dissolved total sugar under (**a**) fencing conditions and (**b**) grazing conditions. The fractions of unexplained variations are not illustrated.

Climatic factors, AGB and plant community composition had exclusive effects on the data matrix of the CP, ADF, NDF, Ash, EE and DTS contents under the fencing conditions and grazing conditions (Figure 4). Climate factors, AGB and plant community composition had exclusive effects on the CP content, NDF content and Ash content under the fencing conditions, and the ADF content, Ash content, EE content and DTS content under the grazing conditions (Figure S2). The SR also had exclusive effects on the NDF content and Ash content under the fencing conditions, and the NDF content, Ash content, EE content and DTS content under the grazing conditions (Figure S2). Climate factors and plant community composition had exclusive effects on the CP content and NDF content under the grazing conditions (Figure S2). Plant community composition had exclusive effects on the ADF content and DTS content under the fencing conditions (Figure S2).

### 2.3. Fencing Effects

**Figure 5:**
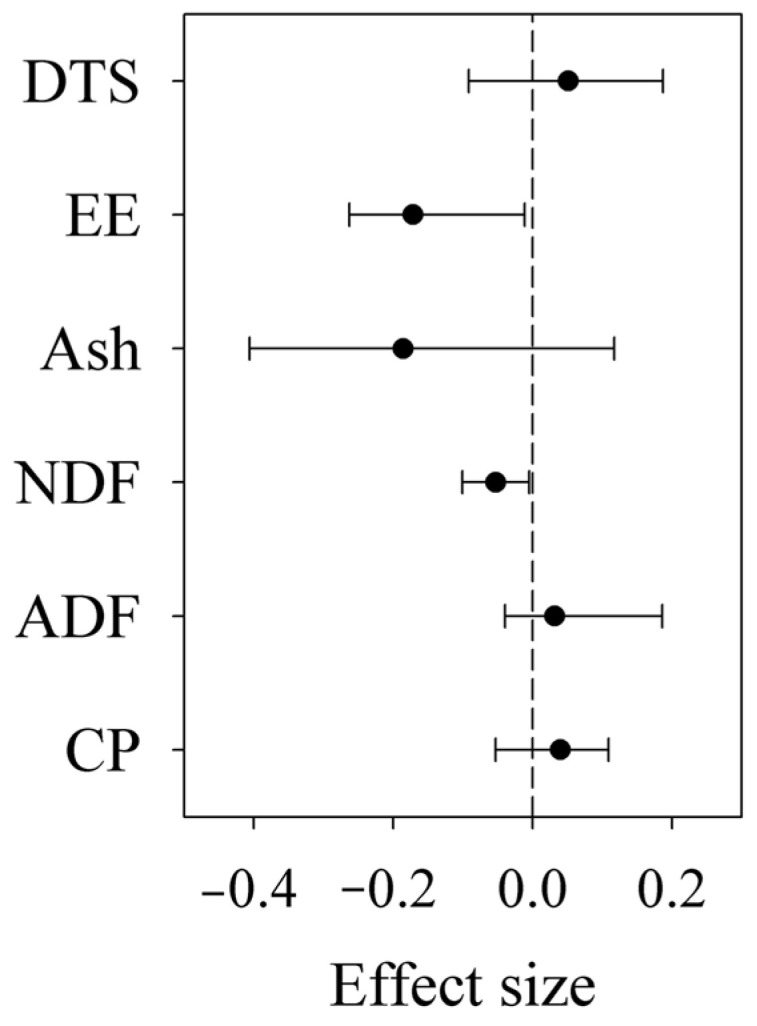
Effect size of fencing on dissolvable total sugar (DTS), crude protein (CP), neutral detergent fiber (NDF), acid detergent fiber (ADF), ether extract (EE) and crude ash (Ash) contents across all the sites. Error bars indicate effect size and 95% bootstrap confidence interval.

Fencing increased AGB by 36.71%, but did not change the plant community composition (*R*^2^ = 0.00, *p* = 0.837) and SR across all the sites (Figure S3). Fencing reduced the NDF content by 5.15% and the EE content by 15.79% across all the sites (Figure 5). However, according to the *Adonis* test on the data matrix of the CP, ADF, NDF, Ash, EE and DTS contents, fencing did not change forage nutrition quality across all the sites (*R*^2^ = 0.04, *p* = 0.389).

**Figure 6:**
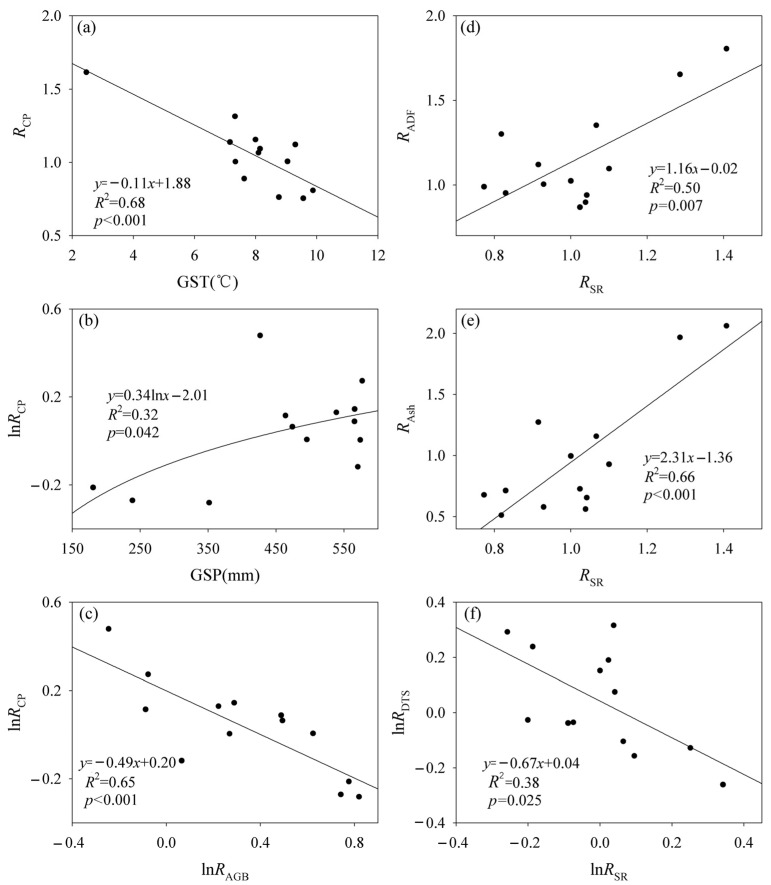
Relationships (**a**) between the effect of fencing on crude protein (*R*_CP_) and growing season temperature (GST), (**b**) between *R*_CP_ and growing season precipitation (GSP), (**c**) between the logarithm of *R*_CP_ (ln*R*_CP_) and the logarithm of the effect of fencing on aboveground biomass (ln*R*_AGB_), (**d**) between the effect of fencing on acid detergent fiber (*R*_ADF_) and the effect of fencing on species richness (*R*_SR_), (**e**) between the effect of fencing on crude ash (*R*_Ash_) and *R*_SR_ and (**f**) between the logarithm of the effect of fencing on dissolved total sugar (ln*R*_DTS_) and the logarithm of *R*_SR_ (ln*R*_SR_).

*R*_AGB_ showed a positive relationship with GST and the data matrix of GST and GSP, and a negative relationship with GSP (Figure S4, Table S4). *R*_CP_ decreased with increasing GST, and the logarithm of *R*_CP_ (ln*R*_CP_) increased with increasing GSP (Figure 6). ln*R*_CP_ decreased with an increasing logarithm of *R*_AGB_ (ln*R*_AGB_) (Figure 6). *R*_SR_ showed positive relationships with *R*_ADF_ and *R*_Ash_, and the logarithm of *R*_SR_ (ln*R*_SR_) showed a negative relationship with the logarithm of *R*_DTS_ (ln*R*_DTS_) (Figure 6). By contrast, *R*_CP_, *R*_ADF_, *R*_NDF_, *R*_Ash_, *R*_EE_ and *R*_DTS_ were not correlated with the effect of fencing on plant community composition (Table S5). The data matrix of *R*_CP_, *R*_ADF_, *R*_NDF_, *R*_Ash_, *R*_EE_ and *R*_DTS_ was correlated with *R*_SR_ (Table S6).

**Figure 7:**
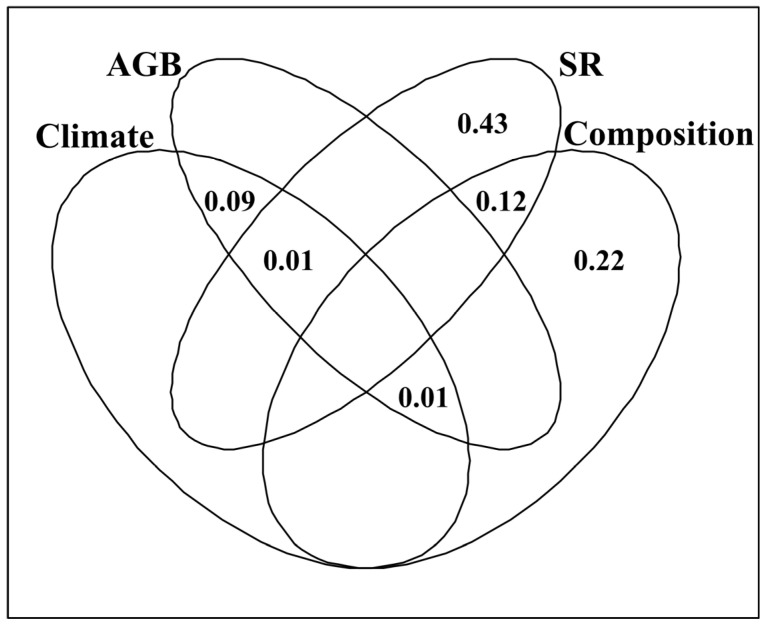
Venn plots of variation partitioning analysis, showing the shared and exclusive effects of climatic factors, and the response ratios of aboveground biomass (AGB), species richness (SR) and community composition to fencing on the data matrix of the response ratio of crude protein, acid detergent fiber, neutral detergent fiber, crude ash, ether extract and dissolved total sugar to fencing. The fractions of unexplained variations are not illustrated.

Plant community composition and SR, rather than AGB and climatic factors, had exclusive effects on the data matrix of *R*_CP_, *R*_ADF_, *R*_NDF_, *R*_Ash_, *R*_EE_ and *R*_DTS_ (Figure 7). Climatic factors and AGB had exclusive effects on *R*_CP_, while SR and community composition had exclusive effects on *R*_NDF_ and *R*_Ash_ (Figure S5). Climatic factors, SR and community composition had exclusive effects on *R*_EE_, while AGB, SR and community composition had exclusive effects on *R*_ADF_ (Figure S5). Only SR had an exclusive effect on *R*_DTS_ (Figure S5). Climatic factors and AGB had shared effects on *R*_CP_, *R*_ADF_, *R*_NDF_, *R*_Ash_, *R*_EE_ and *R*_DTS_ (Figure S5), and the data matrix of *R*_CP_, *R*_ADF_, *R*_NDF_, *R*_Ash_, *R*_EE_ and *R*_DTS_ (Figure 7). Community composition and SR had shared effects on *R*_CP_, *R*_ADF_, *R*_Ash_, *R*_EE_ and *R*_DTS_ (Figure S5), and the data matrix of *R*_CP_, *R*_ADF_, *R*_NDF_, *R*_Ash_, *R*_EE_ and *R*_DTS_ (Figure 7).

## 3. Discussion

The CP content (7.19–17.45%), ADF content (25.56–70.52%), NDF content (50.05–73.49%), Ash content (9.70–34.71%), EE content (1.42–3.44%), and DTS content (1.99–7.65%) in this study were comparable to those of previous observations (CP: 2.71–19.21%, ADF: 9.06–55.39%, NDF: 12.16–76.23%, EE: 0.64–10.90%, Ash: 3.49–21.95%, DTS: 1.24–17.27%) in alpine grasslands on the Tibetan Plateau [5,33,34,35,36,37]. Moreover, the CP content in this study must be able to satisfy the needs of livestock for crude protein [5,38].

### 3.1. Trade-off between Forage Quantity and Nutrition Quality

This study found that AGB was negatively correlated with forage nutrition quality across all the sites, which is in line with previous studies [27,34]. This may be due to the following mechanisms. Plants are generally composed of mechanical tissues and physiologically active non-mechanical tissues. There is generally greater nitrogen and phosphorus, but lower fiber, in physiologically active non-mechanical tissues [39]. By contrast, there is generally lower nitrogen and phosphorus, but greater fiber, in mechanical tissues [39]. With an increase in AGB, mechanical tissues increase but non-mechanical tissues and non-structural carbohydrates (e.g., DTS) decrease [10].

Some studies indicated that species diversity was negatively correlated with the protein content of herbage [8,40]. By contrast, other studies found that species diversity was positively correlated with the CP of herbage, and negatively correlated with the NDF of herbage [41]. Moreover, in an alpine grassland of the Northern Tibet, the NDF content was not significantly correlated with plant species diversity [8]. These inconsistent findings were strengthened by this study, which found that an increase in species diversity may not always increase the nutrition quality of herbage. The nutrition quality of herbage can vary among herbage species and functional groups [2,8]. Grasses may have higher NDF and ADF contents than legumes and forbs, whereas legumes may have higher CP content [7]. Therefore, these contradictory results may be attributed to the extensive variability of species composition.

### 3.2. Climate Change Effects

Warming may reduce the CP content under the fencing conditions (Figure 3a). The Northern Tibet is one of the most sensitive regions to climatic warming [13]. These findings imply that climatic warming may reduce forage nutrition quality of alpine grasslands by reducing the CP content in the Northern Tibet, not considering precipitation change. By contrast, a previous study demonstrated that the effects of experimental warming on the CP content and forage nutrition quality varied with meadow sites and years in the Northern Tibet [5]. These consistent findings may be attributed to one or more of the mechanisms. First, warming can generally increase the ratio of stems to leaves and cell-wall content and amplify the lignification process, but reduce water content of plant tissues [10]. The CP contents in stems may be generally lower than those in leaves [42]. Second, warming may not always increase aboveground plant production in alpine regions [30], which, in turn, may result in different feedbacks to the CP content [41]. Third, the effect of warming on forage nutrition quality can vary with local climate conditions and warming magnitude [2,5]. Spatial asymmetrical warming may homogenize plant production and diversity [43].

Increased precipitation cannot necessarily increase the CP content, and there was optimal precipitation for the CP content under the fencing conditions (Figure 3b). This finding is similar to those of some previous studies [2,8]. This phenomenon may be attributed to one or more mechanisms. First, increased precipitation may generally result in lower temperature [30], which, in turn, may alter the CP content [44]. Second, increased precipitation can have non-linear effects on plant production, α-diversity and community composition [30], which, in turn, can cause different changes in the CP content. Third, increased precipitation may have cumulative effects on plant production, α-diversity and community composition [30].

Increased precipitation can reduce the NDF content under the fencing conditions (Figure 3c), which is consistent with the conclusions found in a previous study [45]. This finding may be attributed to one or more mechanisms. First, decreased precipitation may suppress the generation of new tissues and accelerate the process of lignification. In contrast, increased precipitation may suppress the accumulation of fibrous and less-digestible material. Second, a precipitation-induced change in SR may mainly affect forbs’ SR rather than grasses’ SR, and forbs may have lower NDF content than grasses [8,41].

This study found that precipitation had stronger effects on the CP and NDF contents than did air temperature under the fencing conditions, which was in line with the fact that both SR and AGB had closer relationships with GSP than GST [13]. This finding is in line with the results found in some previous studies [5,45,46]. Therefore, increased precipitation may have stronger effects on forage nutrition quality in alpine grasslands than climate warming does under future climate change scenarios.

### 3.3. Fencing Effects

This study found that fencing did not result in better nutrition quality in alpine grasslands across all the sites (Figure 5), which may be explained by one or more of the following mechanisms. First, grasslands with higher CP, EE, DTS and Ash contents, and lower ADF and NDF contents, generally have better nutrition quality [5,27]. On one hand, fencing reduced both the EE and NDF contents (Figure 5). On the other hand, the response of SR to fencing showed different relationships to that of the ADF, Ash and DTS contents (Figure 2). Second, the selective intake of pasture by grazing animals may affect the nutrition quality of herbage by changing plant diversity and community composition. However, consistent with some previous studies [47,48], fencing did not affect SR and community composition. Third, similarly to some previous studies [48,49], fencing increased AGB. However, the response of AGB to fencing had a negligible exclusive correlation with that of forage nutrition quality (Figure 7). Fourth, on one hand, grazing may result in plant compensatory growth and new tissues. New organization may have better nutrition quality than old organization [50,51,52]. On the other hand, fencing may result in increases in herbage height [48]. With an increase in herbage height, the forage nutrition quality of grasslands may reduce [53,54].

### 3.4. Interactive Effects of Fencing and Climate Change

The CP content was more responsive to fencing in colder and wetter scenarios (Figure 6). This finding was in line with a previous study, which demonstrated that the response of forage nutrition quality to fencing increased with increasing precipitation across the Tibetan grassland ecosystems [2]. By contrast, AGB was more responsive to fencing in warmer and drier scenarios. These findings were in line with the negative relationship between the response of CP content to fencing and the response of AGB to fencing. On one hand, climatic conditions vary spatially and temporally in the Northern Tibet [13]. Therefore, the effects of fencing on the CP content and AGB can change spatially and temporally. This finding strengthened a previous study [48] which found that the response of grassland productivity, rather than the nutrition quality of grassland, to fencing may change spatially and temporally. On the other hand, fencing can only be set up in a certain range of climatic conditions (e.g., GSP: 427.89–661.12 mm; GST: 5.19–8.44 °C in this study) to achieve the goals of simultaneously restoring forage productivity and nutrition quality in grassland ecological systems. When climatic conditions are beyond this certain range, fencing may only achieve the goal of restoring grassland productivity or forage nutrition quality. Therefore, fencing with a dynamic classification mode may be needed to achieve the restoration of degraded grasslands and grassland management in the Northern Tibet.

Fencing altered the temperature and/or precipitation sensitivity of forage nutrition quality (Figure 3). This finding strengthened some previous studies, which found that fencing altered the temperature and/or precipitation sensitivity of plant species diversity, forage nutrition quality and storge across grassland ecological systems on the Tibetan Plateau [1,2,13]. This phenomenon may be attributed to one or more mechanisms. First, grazing may increase environmental temperature and, in turn, alter the energy balance due to lower vegetation coverage. An increase in temperature can, in turn, generally reduce temperature sensitivity in plant productivity [55]. Second, fencing can increase AGB and, in turn, change the precipitation sensitivity of AGB [48]. Third, forage nutrition quality was also closely correlated with both SR and community composition [10]. Both SR and community composition had closer relationships with climatic variables under the grazing conditions than fencing conditions [47].

This study also implied that fencing altered the spatial distribution patterns of forage nutrition quality in the Northern Tibet grasslands, which is in line with some previous studies [1,2]. A recent study ascribed this finding to the effects of grazing on plant phenology, species diversity and composition, and soil nutrition, etc., varied with geographic position [2]. Moreover, fencing can also alter the spatial distribution pattern of soil pH in grasslands on the Tibetan Plateau [32]. Soil pH can be also correlated with forage nutrition quality in the Northern Tibet grasslands [8]. Third, the grazing seasons are different in different grassland regions, and the effects of winter grazing and summer grazing on forage nutrition quality are different [8]. Fourth, grazing intensity can also be correlated with the effect of fencing on forage nutrition quality [37]. Different grassland regions can generally have different grazing intensities [2,37].

## 4. Materials and Methods

### 4.1. AGB Sampling and Forage Nutrition Component Analyses

The AGB of 13 sampling sites in alpine grasslands over the Northern Tibet (Table S7; 4 alpine meadows, 3 alpine steppes, 6 alpine desert steppes) were collected in 2018. At each sampling site, four 0.50 m × 0.50 m (meadows) or 1.00 m × 1.00 m (steppes) quadrats were established under fencing and grazing conditions, respectively. Meanwhile, species richness (SR) was investigated for each quadrat. Plants’ aboveground parts were clipped, dried at 65 °C for 48 h and weighted for each species. The aboveground parts of all the species were mixed, crushed and passed through a sieve (0.4 mm) before nutritional component analyses for each quadrat. The Soxhlet extraction, Kjeldahl, complete combustion, anthrone-based and Van Soest methods were used to analyze the ether extract (EE), CP, crude ash (Ash), dissolvable total sugar (DTS) and ADF/NDF contents, respectively [2,56].

### 4.2. Statistical Analyses

We calculated proportional biomass of each species out of the total biomass (*P_i_*) in each quadrat. The data matrix of *P_i_* was used to reflect the plant community composition in each quadrat [56]. An independent-samples *t*-test was used to examine the differences in the contents of the six nutritional quality variables between the grazing and fencing conditions for each site using SPSS 16.0. We calculated the ratio of CP (*R*_CP_), EE (*R*_EE_), ADF (*R*_ADF_), NDF (*R*_NDF_), Ash (*R*_Ash_) and DTS (*R*_DTS_) between the fencing and grazing conditions for each sampling site [2]. We used METAWIN 2.1 (Sinauer Associates Inc., Sunderland, MA, USA) to examine whether these ratios were significant (detailed information shown in the supporting materials). We used permutational multivariate analysis of variance (*adonis* test) (vegan package) to examine the differences in the data matrix of plant community composition and data matrix of forage nutritional quality between the fencing and grazing conditions. We used mantel tests (vegan package) to analyze the correlations of CP, ADF, NDF, Ash, EE and DTS, the data matrix of nutritional quality, the data matrix of plant community composition, AGB and SR with the data matrix of GST and GSP. The *adonis* and mantel analyses were performed using R 3.6.1. We also used SPSS 16.0 to perform the regression analysis.

## 5. Conclusions

This study investigated the response of forage nutrition quality to climate change and fencing in grasslands of the Northern Tibetan Plateau. Overall, fencing reduced both the EE and NDF contents by 15.79% and 5.15% across all the sites, respectively. The effect of fencing on the CP content decreased and increased with temperature and precipitation, respectively. The response of the CP content to fencing decreased with the response of AGB to fencing. The response of SR to fencing increased and decreased with the response of the ADF and DTS contents to fencing, respectively. Compared to the response of AGB to fencing, the responses of SR and community composition to fencing had greater effects on the response of forage nutrition quality to fencing. The CP content decreased exponentially with increasing temperature (*R*^2^ = 0.08), and showed a quadratic relationship with precipitation (*R*^2^ = 0.24) under the fencing conditions. By contrast, the CP content increased exponentially with increasing temperature under the grazing conditions. The NDF content decreased logarithmically with precipitation under the fencing and grazing conditions, respectively, but the decreased magnitude was greater under the fencing conditions than the grazing conditions. The ADF content increased with increasing precipitation under the grazing conditions, but there was a no relationship under the fencing conditions. The CP and DTS contents decreased with increasing AGB under the fencing and grazing conditions, respectively. By contrast, the ADF content increased with increasing AGB under the grazing conditions. The CP content decreased with increasing SR under the grazing conditions. By contrast, the ADF content increased with increasing SR under the fencing and grazing conditions, respectively.

The findings observed by this study can provide some guidance for the restoration and management of degraded grasslands, at least for areas of the Northern Tibet. First, the local climate conditions should be taken into account when the fencing is placed because the CP content can be more responsive to fencing in colder and wetter grasslands of the Northern Tibet. Second, climate change does not always have adverse effects on forage nutrition quality. Moreover, we should pay more attention to the effects of precipitation change on forage nutrition quality than those of climate warming. Third, we should focus on not only grassland productivity and species diversity, but also forage quality, when evaluating the effects of climate change and fencing on grasslands. The trade-off relationship between forage nutrition quality and quantity should be considered when fencing is set up. Fourth, we should pay more attention to the effects of fencing-induced changes in plant species α-diversity and community composition on forage nutrition quality, rather than the effects of fencing-induced changes in AGB on forage nutrition quality.

## Data Availability

The data used in this study are available upon request from the corresponding author.

## Supplementary Materials

The following supporting information can be downloaded at: https://www.mdpi.com/article/10.3390/plants12183182/s1, Figure S1: Relationships (a) between species richness (SR) and growing season precipitation (GSP) under fencing conditions, (b) between aboveground biomass (AGB) and GSP under fencing conditions, (c) between SR and GSP under grazing conditions, and (d) between AGB and GSP under grazing conditions; Figure S2: Venn plots of variation partitioning analysis, showing the shared and exclusive effects of climatic factors, aboveground biomass (AGB), species richness (SR) and community composition on (a, b) crude protein, (c, d) acid detergent fiber, (e, f) neutral detergent fiber, (g, h) crude ash, (i, j) ether extract and (k,l) dissolved total sugar under (a, c, e, g, i, k) fencing and (b, d, f, h, j, l) grazing conditions. The fraction of unexplained variations are not illustrated; Figure S3: Effect size of fencing on aboveground biomass (AGB) and species richness (SR) across all the sites. Error bars indicate effect size and 95% bootstrap confidence interval; Figure S4: Relationships (a) between the effect of fencing on aboveground biomass (*R*_AGB_) and growing season temperature (GST), and (b) between the *R*_AGB_ and growing season precipitation (GSP); Figure S5: Venn plots of variation partitioning analysis, showing the shared and exclusive effects of climatic factors, aboveground biomass (AGB), species richness (SR) and community composition on (a) the response ratio of crude protein to fencing, (b) the response ratio of acid detergent fiber to fencing, (c) the response ratio of neutral detergent fiber to fencing, (d) the response ratio of crude ash to fencing, (e) the response ratio of ether extract to fencing and (f) the response ratio of dissolved total sugar to fencing. The fraction of unexplained variations are not illustrated; Table S1: Relationships between plant community composition and crude protein (CP), acid detergent fiber (ADF), neutral detergent fiber (NDF), crude ash (Ash), ether extract (EE), and dissolved total sugar (DTS). All the correlations are based on the mantel test; Table S2: Relationships between the data matrix of the six nutrition component variables (i.e., crude protein, acid detergent fiber, neutral detergent fiber, crude ash, ether extract and dissolved total sugar) and growing season temperature (GST), growing season precipitation (GSP), aboveground biomass (AGB), species richness (SR) and plant community composition. All the correlations are based on the mantel test; Table S3: Relationships between the data matrix of growing season temperature and precipitation, and aboveground biomass (AGB), species richness (SR), plant community composition, crude protein (CP), acid detergent fiber (ADF), neutral detergent fiber (NDF), crude ash (Ash), ether extract (EE), dissolved total sugar (DTS) and the data matrix of the six nutrition component variables. All the correlations are based on the mantel test; Table S4: Relationships between the data matrix of growing season temperature and precipitation, and the fencing effect on aboveground biomass (*R*_AGB_), species richness (*R*_SR_), plant community composition, crude protein (*R*_CP_), acid detergent fiber (*R*_ADF_), neutral detergent fiber (*R*_NDF_), crude ash (*R*_Ash_), ether extract (*R*_EE_), dissolved total sugar (*R*_DTS_) and the data matrix of *R*_CP_, *R*_ADF_, *R*_NDF_, *R*_Ash_, *R*_EE_ and *R*_DTS_. All the correlations are based on the mantel test; Table S5: Relationships between the effect of fencing on plant community composition and that of crude protein (*R*_CP_), acid detergent fiber (*R*_ADF_), neutral detergent fiber (*R*_NDF_), crude ash (*R*_Ash_), ether extract (*R*_EE_), and dissolved total sugar (*R*_DTS_). All the correlations are based on the mantel test; Table S6: Relationships between the data matrix of the fencing effects on the six nutrition component variables (i.e., crude protein, acid detergent fiber, neutral detergent fiber, crude ash, ether extract and dissolved total sugar) and growing season temperature (GST), growing season precipitation (GSP), the fencing effect on aboveground biomass (AGB), the fencing effect on species richness (SR) and the fencing effect on plant community composition. All the correlations are based on the mantel test; Table S7: Comparison of the contents of crude protein, acid detergent fiber, neutral detergent fiber, crude ash, ether extract and dissolvable total sugar between fencing and grazing conditions (Mean ± SD).
