# Supplementary material for: Effects of Climate Change and Fencing on Forage Nutrition Quality of Alpine Grasslands in the Northern Tibet
Source: Plants (Basel). 2023 Sep 6;12(18):3182. doi: 10.3390/plants12183182 (PMC10538120; doi:10.3390/plants12183182)
Supplement: Supplementary file 1 [file plants-12-03182-s001.zip › plants-2587772-supplementary.pdf]

Materials and Methods

Mean annual temperature is -2.3–1.2 °C and mean annual precipitation is <100–>450 mm in the Northern Tibetan Plateau. There are *Kobresia*-dominant alpine meadows, *Stipa*-dominant alpine steppes, and *Stipa*-dominant alpine desert steppes from east to west across the Northern Tibetan Plateau. Fencing was conducted in autumn 2006 or spring 2007, and each fencing is at least 25 hm^2. Alpine grasslands outside the fencings are freely grazed by domestic animals (e.g. domestic sheep, goats and yaks) and wild animals (e.g. Tibetan antelopes and kiangs) throughout the year or during the cold season. The grazing intensity was approximately 0.50–1.00 sheep units ha^{-1} in alpine meadows, 0.25–0.33 sheep units ha^{-1} in alpine steppes and 0.13–0.17 sheep units ha^{-1} in alpine desert steppes. The grazing quadrats were generally 1000 m distance from the edge of fencing.

$$R = \overline{X_t} / \overline{X_c} \quad (1)$$

where $\overline{X_c}$ and $\overline{X_t}$ are the content of nutrition component variables (i.e. crude protein (CP), ether extract (EE), acid detergent fiber (ADF), neutral detergent fiber (NDF), crude ash (Ash) and dissolvable sugar (DTS)) under grazing and fencing conditions, respectively.

A fixed effects model was used to examine whether R_{CP}, R_{EE}, R_{ADF}, R_{NDF}, R_{Ash} and R_{DTS} was significant, respectively. The R_{CP}, R_{EE}, R_{ADF}, R_{NDF} and R_{Ash} was statistically significant if the 95% bootstrap CI did not cover zero, respectively.

The natural logarithm of response ratio (*R*) and the inverse of pooled variance ($1/v$) was as effect size and weighting factor (w), respectively.

$$v = \frac{S_t^2}{n_t \overline{X_t^2}} + \frac{S_c^2}{n_c \overline{X_c^2}} \quad (2)$$

where S_c^2 and S_t^2 are standard deviations under grazing and fencing conditions, respectively; n_c and n_t are numbers of replication under grazing and fencing conditions, respectively.

Mean effect size ($\overline{\ln R}$) for all observations was calculated,

$$\overline{\ln R} = \frac{\sum_{i=1}^{m} w_i \ln R_i}{\sum_{i=1}^{m} w_i} \quad (3)$$

where $\ln R_i$ and w_i are $\ln R$ and w of the *i*th observation, respectively.

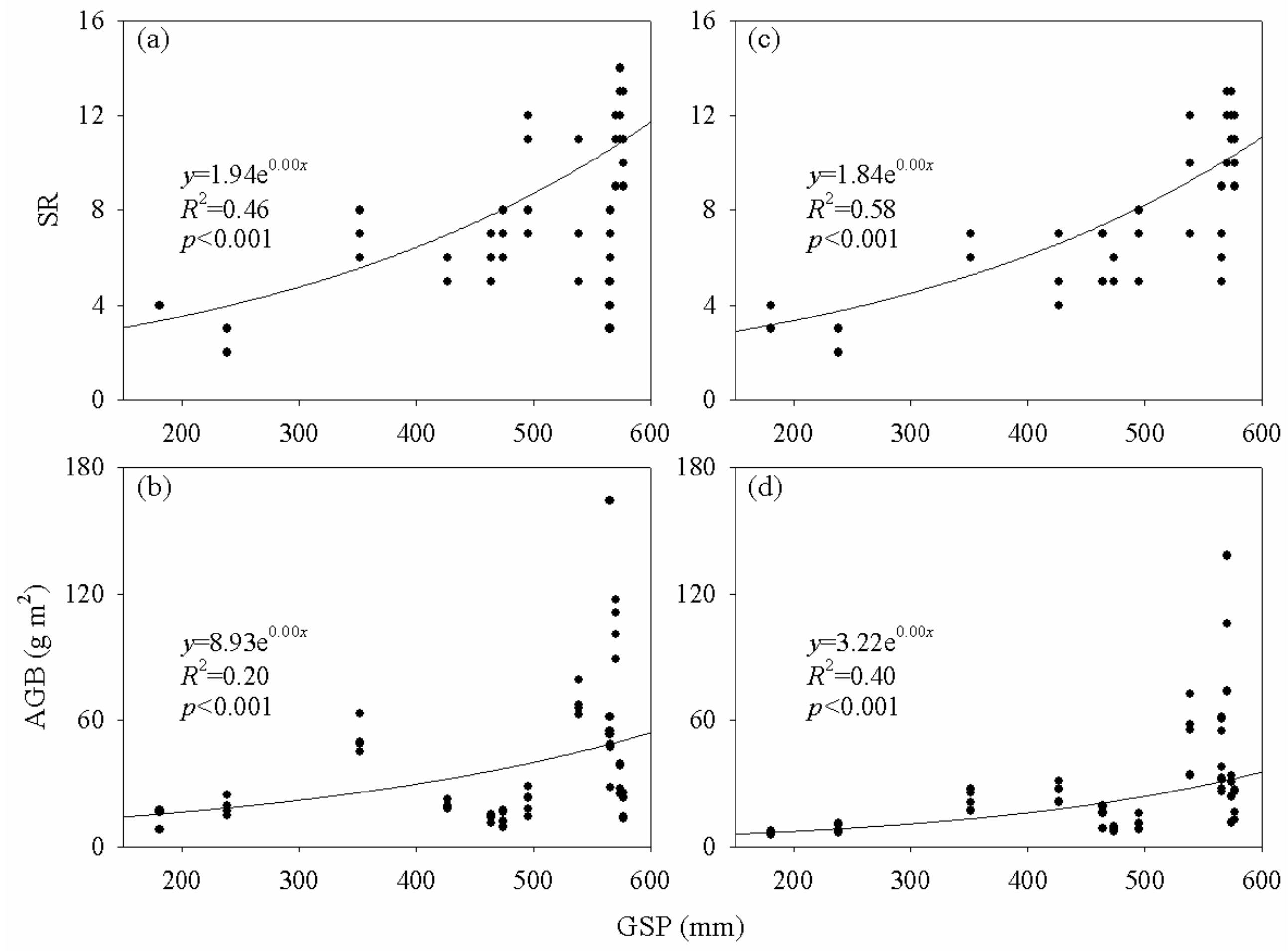

Figure S1. Relationships (a) between species richness (SR) and growing season precipitation (GSP) under fencing conditions, (b) between aboveground biomass (AGB) and GSP under fencing conditions, (c) between SR and GSP under grazing conditions, and (d) between AGB and GSP under grazing conditions.

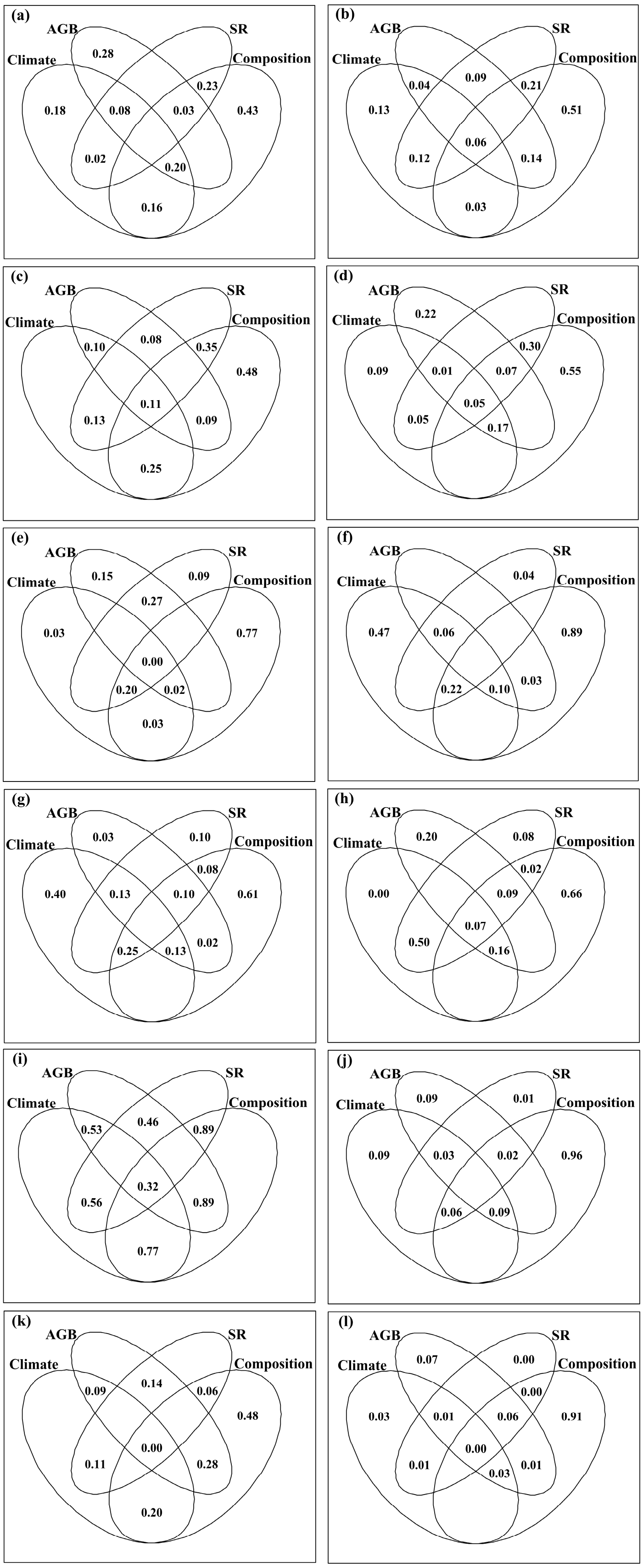

Figure S2. Venn plots of variation partitioning analysis, showing the shared and exclusive effects of climatic factors, aboveground biomass (AGB), species richness (SR) and community composition on (a, b) crude protein, (c, d) acid detergent fiber, (e, f) neutral detergent fiber, (g, h) crude ash, (i, j) ether extract and (k,l) dissolved total sugar under (a, c, e, g, i, k) fencing and (b, d, f, h, j, l) grazing conditions. The fraction of unexplained variations are not illustrated.

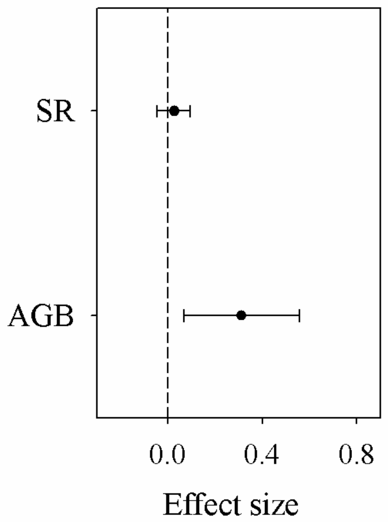

Figure S3. Effect size of fencing on aboveground biomass (AGB) and species richness (SR) across all the sites. Error bars indicate effect size and 95% bootstrap confidence interval.

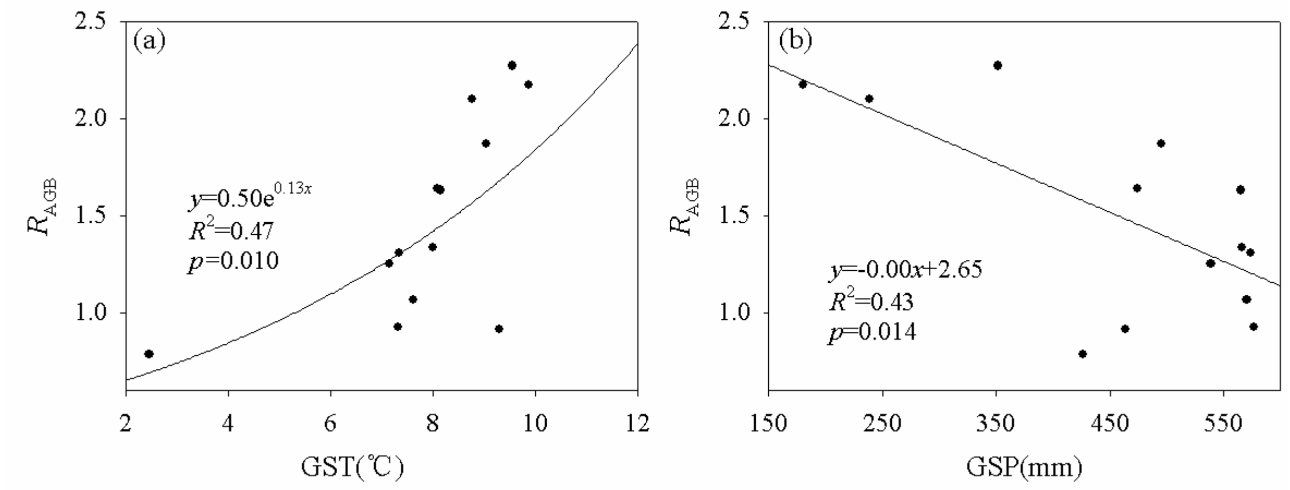

Figure S4. Relationships (a) between the effect of fencing on aboveground biomass (R_{AGB}) and growing season temperature (GST), and (b) between the R_{AGB} and growing season precipitation (GSP).

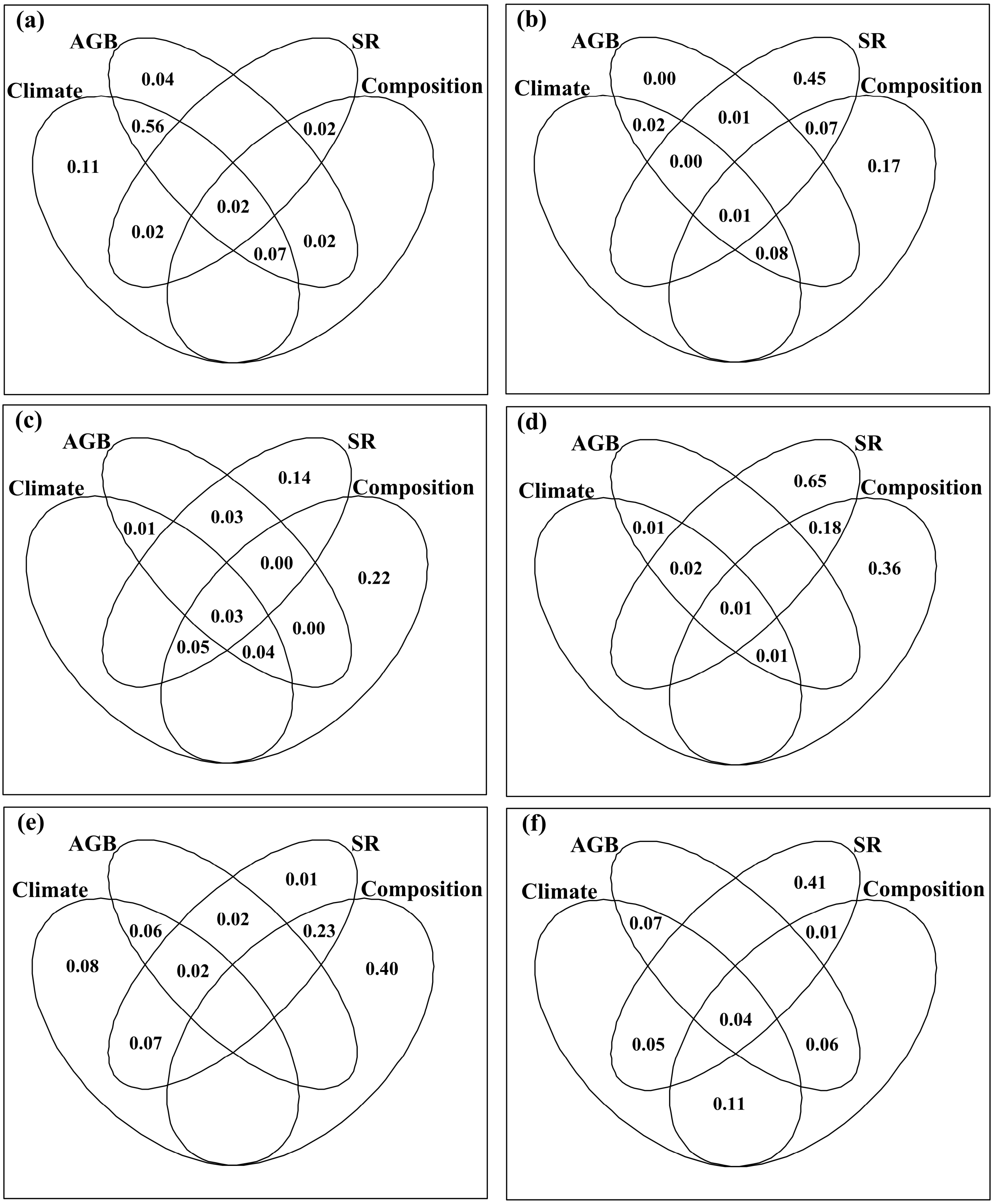

Figure S5. Venn plots of variation partitioning analysis, showing the shared and exclusive effects of climatic factors, aboveground biomass (AGB), species richness (SR) and community composition on (a) the response ratio of crude protein to fencing, (b) the response ratio of acid detergent fiber to fencing, (c) the response ratio of neutral detergent fiber to fencing, (d) the response ratio of crude ash to fencing, (e) the response ratio of ether extract to fencing and (f) the response ratio of dissolved total sugar to fencing. The fraction of unexplained variations are not illustrated.

Table S1. Relationships between plant community composition and crude protein (CP), acid detergent fiber (ADF), neutral detergent fiber (NDF), crude ash (Ash), ether extract (EE), and dissolved total sugar (DTS). All the correlations are based on the mantel test.

Land use type	Variables	*R*	*p*
Fencing	CP	0.05	0.094
	**ADF**	**0.11**	**0.007**
	**NDF**	**0.07**	**0.034**
	Ash	0.05	0.082
	EE	-0.01	0.533
	**DTS**	**0.07**	**0.024**
Grazing	**CP**	**0.12**	**0.002**
	**ADF**	**0.15**	**0.001**
	NDF	0.01	0.418
	**Ash**	**0.12**	**0.008**
	**EE**	**0.14**	**0.001**
	DTS	0.02	0.229

Table S2. Relationships between the data matrix of the six nutrition component variables (i.e., crude protein, acid detergent fiber, neutral detergent fiber, crude ash, ether extract and dissolved total sugar) and growing season temperature (GST), growing season precipitation (GSP), aboveground biomass (AGB), species richness (SR) and plant community composition. All the correlations are based on the mantel test.

Land use type	Variables	*R*	*p*
Fencing	GST	-0.11	0.915
	GSP	-0.02	0.647
	AGB	-0.12	0.963
	**SR**	**0.35**	**0.001**
	**Plant community composition**	**0.15**	**0.001**
Grazing	GST	-0.06	0.675
	GSP	0.02	0.401
	AGB	-0.05	0.695
	**SR**	**0.25**	**0.001**
	**Plant community composition**	**0.18**	**0.001**

Table S3. Relationships between the data matrix of growing season temperature and precipitation, and aboveground biomass (AGB), species richness (SR), plant community composition, crude protein (CP), acid detergent fiber (ADF), neutral detergent fiber (NDF), crude ash (Ash), ether extract (EE), dissolved total sugar (DTS) and the data matrix of the six nutrition component variables. All the correlations are based on the mantel test.

Land use type	Variable	*R*	*p*
Fencing	AGB	-0.01	0.419
	SR	**0.30**	**0.001**
	Plant community composition	**0.40**	**0.001**
	CP	**0.15**	**0.010**
	ADF	-0.09	0.901
	NDF	**0.16**	**0.018**
	Ash	-0.08	0.926
	EE	0.04	0.271
	DTS	-0.10	0.976
	Nutrition component matrix	-0.03	0.630
Grazing	AGB	0.05	0.234
	SR	**0.35**	**0.001**
	Plant community composition	**0.41**	**0.001**
	CP	-0.09	0.899
	ADF	-0.00	0.440
	NDF	0.10	0.082
	Ash	-0.01	0.467
	EE	-0.03	0.525
	DTS	-0.07	0.870
	Nutrition component matrix	0.01	0.382

Table S4. Relationships between the data matrix of growing season temperature and precipitation, and the fencing effect on aboveground biomass (R_{AGB}), species richness (R_{SR}), plant community composition, crude protein (R_{CP}), acid detergent fiber (R_{ADF}), neutral detergent fiber (R_{NDF}), crude ash (R_{Ash}), ether extract (R_{EE}), dissolved total sugar (R_{DTS}) and the data matrix of R_{CP}, R_{ADF}, R_{NDF}, R_{Ash}, R_{EE} and R_{DTS}. All the correlations are based on the mantel test.

Variable	*R*	*p*
R_{AGB}	**0.48**	**0.004**
R_{SR}	-0.12	0.715
Plant community composition	-0.12	0.700
R_{CP}	0.28	0.114
R_{ADF}	0.04	0.281
R_{NDF}	0.02	0.388
R_{Ash}	-0.10	0.642
R_{EE}	-0.05	0.532
R_{DTS}	-0.10	0.738
Data matrix of R_{CP},R_{ADF}, R_{NDF}, R_{Ash}, R_{EE} and R_{DTS}	-0.07	0.581

Table S5. Relationships between the effect of fencing on plant community composition and that of crude protein (R_{CP}), acid detergent fiber (R_{ADF}), neutral detergent fiber (R_{NDF}), crude ash (R_{Ash}), ether extract (R_{EE}), and dissolved total sugar (R_{DTS}). All the correlations are based on the mantel test.

Variable	*R*	*p*
R_{CP}	0.16	0.285
R_{ADF}	0.08	0.329
R_{NDF}	0.20	0.159
R_{Ash}	0.08	0.382
R_{EE}	0.11	0.257
R_{DTS}	-0.10	0.758

Table S6. Relationships between the data matrix of the fencing effects on the six nutrition component variables (i.e. crude protein, acid detergent fiber, neutral detergent fiber, crude ash, ether extract and dissolved total sugar) and growing season temperature (GST), growing season precipitation (GSP), the fencing effect on aboveground biomass (AGB), the fencing effect on species richness (SR) and the fencing effect on plant community composition. All the correlations are based on the mantel test.

Variables	*R*	*p*
GST	-0.02	0.357
GSP	-0.07	0.586
R_{AGB}	0.03	0.365
R_{SR}	**0.67**	**0.005**
Plant community composition	0.15	0.236

Table S7. Comparison of the contents of crude protein, acid detergent fiber, neutral detergent fiber, crude ash, ether extract and dissolvable total sugar between fencing and grazing conditions (Mean±SD).

Site	Lat(°)	Lon(°)	Land use type	Crude protein(%)	Acid detergent fiber(%)	Neutral detergent fiber(%)	Crude ash(%)	Ether extract(%)	Dissolvable total sugar(%)	Adonis test	
										R^2	*p*
Bang Ai(BA)	32.29	91.84	Grazing	**12.24±0.67 b**	40.80±3.92	63.00±9.53	**17.42±0.76 a**	2.04±0.46	2.69±0.24	0.17	0.375
			Fencing	**13.93±1.18 a**	38.84±4.41	57.67±7.79	**12.44±3.03 b**	2.44±2.09	3.41±1.36		
Guo Zu(GZ)	31.72	91.81	Grazing	10.87±1.36	43.94±5.13	58.66±2.06	11.67±1.41	2.86±1.04	3.40±0.56	0.23	0.142
			Fencing	9.67±0.63	49.25±2.57	56.29±3.99	14.87±6.11	2.29±0.81	3.27±0.64		
Na Ma Qie(NMQ)	31.58	91.46	Grazing	11.38±1.37	34.49±4.2	**73.49±1.65 a**	19.82±6.69	2.28±0.33	5.03±1.15	0.50	**0.026**
			Fencing	13.15±1.44	34.64±4.64	**63.33±2.63 b**	11.47±3.83	3.06±1.95	4.86±0.48		
Na Qu(NQ)	31.64	92.01	Grazing	12.12±1.85	39.56±3.97	62.39±2.67	15.41±6.55	2.08±0.14	1.99±0.35	0.16	0.339
			Fencing	13.24±0.90	39.14±5.02	59.89±3.32	10.45±1.97	1.63±1.05	2.67±1.15		
Ga Cuo(GC)	33.70	88.24	Grazing	**9.03±2.01 b**	34.50±2.98	58.85±5.60	12.51±5.06	2.48±0.21	4.35±0.61	0.16	0.382
			Fencing	**14.59±2.35 a**	37.83±3.00	59.28±6.90	11.62±3.70	1.96±0.87	3.72±0.57		
Pu Bao(PB)	31.39	90.31	Grazing	10.18±0.99	**70.52±1.88 a**	62.93±4.12	**29.95±6.24 a**	2.24±3.17	2.04±0.32	0.46	0.053
			Fencing	10.22±1.03	**66.30±2.38 b**	59.06±4.48	**19.62±3.81 b**	1.72±1.21	2.20±0.36		
Xiong Mei(XM)	31.31	88.91	Grazing	8.81±3.06	40.39±6.57	56.63±2.84	29.68±8.73	1.42±1.04	4.02±1.31	0.18	0.263
			Fencing	11.58±2.43	35.08±5.35	54.24±8.67	21.59±9.49	2.11±0.87	4.86±0.89		

Dong Cuo(DC)	31.99	84.83	Grazing	9.52±1.86	**49.95±3.00 a**	62.42±4.35	**34.71±5.60 a**	1.99±0.29	**2.25±0.10b**	0.70	**0.022**
			Fencing	7.19±0.22	**44.85±2.43 b**	63.97±0.44	**19.49±2.56 b**	2.11±1.13	**3.08±0.39 a**		
E Jiu Si(EJS)	32.08	86.90	Grazing	16.36±0.91	**25.56±1.09 b**	60.17±2.26	**12.84±1.12 b**	2.88±0.09	4.73±1.09	0.68	**0.028**
			Fencing	17.45±3.10	**42.26±6.16 a**	55.23±5.07	**25.26±7.39 a**	1.88±1.41	4.17±1.31		
E Jiu Yi(EJY)	31.95	86.89	Grazing	14.90±1.70	33.80±3.41	58.63±5.21	14.91±6.19	2.79±0.34	3.45±0.44	0.05	0.854
			Fencing	16.72±0.77	34.61±2.94	56.69±6.52	14.86±4.25	2.16±0.48	4.01±1.18		
Ni Ma(NM)	31.80	87.29	Grazing	13.41±2.01	**26.63±4.67 b**	50.05±2.62	11.94±4.03	2.65±0.55	7.65±0.79	0.56	**0.028**
			Fencing	13.50±3.04	**48.06±11.58a**	52.75±5.5	24.62±10.29	1.80±0.67	5.90±1.21		
Wen Bu(WB)	32.22	82.54	Grazing	**12.21±1.50 a**	**32.49±2.33 b**	67.33±3.29	**18.94±6.34 a**	2.85±0.37	3.27±0.75	0.62	**0.035**
			Fencing	**9.32±1.70 b**	**42.28±1.83 a**	68.30±1.98	**9.70±2.09 b**	3.44±1.69	3.18±0.37		
Xiong Ba(XB)	32.02	81.54	Grazing	11.94±1.40	**30.01±1.78 b**	69.80±1.74	13.01±1.47	2.80±0.08	4.71±0.45	0.50	**0.026**
			Fencing	9.66±2.66	**40.59±6.57 a**	64.37±4.89	15.06±3.68	1.97±0.94	4.25±0.78		

Different letters indicate significant differences between fencing and grazing conditions at $p<0.05$.
